# rAAV-compatible MiniPromoters for restricted expression in the brain and eye

**DOI:** 10.1186/s13041-016-0232-4

**Published:** 2016-05-10

**Authors:** Charles N. de Leeuw, Andrea J. Korecki, Garrett E. Berry, Jack W. Hickmott, Siu Ling Lam, Tess C. Lengyell, Russell J. Bonaguro, Lisa J. Borretta, Vikramjit Chopra, Alice Y. Chou, Cletus A. D’Souza, Olga Kaspieva, Stéphanie Laprise, Simone C. McInerny, Elodie Portales-Casamar, Magdalena I. Swanson-Newman, Kaelan Wong, George S. Yang, Michelle Zhou, Steven J. M. Jones, Robert A. Holt, Aravind Asokan, Daniel Goldowitz, Wyeth W. Wasserman, Elizabeth M. Simpson

**Affiliations:** Centre for Molecular Medicine and Therapeutics at the Child & Family Research Institute, University of British Columbia, 950 W 28 Ave, Vancouver, BC V5Z 4H4 Canada; Department of Medical Genetics, University of British Columbia, Vancouver, BC V6H 3N1 Canada; Gene Therapy Centre, University of North Carolina, Chapel Hill, NC 27599 U.S.A.; Canada’s Michael Smith Genome Sciences Centre, British Columbia Cancer Agency, Vancouver, BC V5Z 4S6 Canada; Department of Molecular Biology and Biochemistry, Simon Fraser University, Burnaby, BC V5A 1S6 Canada; Department of Psychiatry, University of British Columbia, Vancouver, BC V6T 2A1 Canada

**Keywords:** rAAV Gene therapy, Raphe nuclei, Purkinje cells, Retina, Cornea

## Abstract

**Background:**

Small promoters that recapitulate endogenous gene expression patterns are important for basic, preclinical, and now clinical research. Recently, there has been a promising revival of gene therapy for diseases with unmet therapeutic needs. To date, most gene therapies have used viral-based ubiquitous promoters–however, promoters that restrict expression to target cells will minimize off-target side effects, broaden the palette of deliverable therapeutics, and thereby improve safety and efficacy. Here, we take steps towards filling the need for such promoters by developing a high-throughput pipeline that goes from genome-based bioinformatic design to rapid testing in vivo.

**Methods:**

For much of this work, therapeutically interesting Pleiades MiniPromoters (MiniPs; ~4 kb human DNA regulatory elements), previously tested in knock-in mice, were “cut down” to ~2.5 kb and tested in recombinant adeno-associated virus (rAAV), the virus of choice for gene therapy of the central nervous system. To evaluate our methods, we generated 29 experimental rAAV2/9 viruses carrying 19 different MiniPs, which were injected intravenously into neonatal mice to allow broad unbiased distribution, and characterized in neural tissues by X-gal immunohistochemistry for icre, or immunofluorescent detection of GFP.

**Results:**

The data showed that 16 of the 19 (84 %) MiniPs recapitulated the expression pattern of their design source. This included expression of: Ple67 in brain raphe nuclei; Ple155 in Purkinje cells of the cerebellum, and retinal bipolar ON cells; Ple261 in endothelial cells of brain blood vessels; and Ple264 in retinal Müller glia.

**Conclusions:**

Overall, the methodology and MiniPs presented here represent important advances for basic and preclinical research, and may enable a paradigm shift in gene therapy.

**Electronic supplementary material:**

The online version of this article (doi:10.1186/s13041-016-0232-4) contains supplementary material, which is available to authorized users.

## Background

Interest in the identification of small promoters for recapitulating unique gene expression patterns is longstanding, as such promoters are widely used in basic and preclinical research [1–6]. Recently, there has been a promising revival of gene therapy for diseases with unmet treatment needs [7]. Most *avant garde* of these are Imlygic® and Glybera®, virus-based gene therapies approved for marketing by the U.S. Food and Drug Administration in 2015, and the European Medicines Agency in 2013, respectively. To date, these and most gene therapies have used viral-based ubiquitous promoters. However, it has been recognized by leaders in the field that “a more deliberate process of promoter selection may be beneficial in the long run by determining the minimum promoter activity that is necessary to tailor AAV-mediated gene therapy to a particular neurological disease.” [8]. It is anticipated that promoters that restrict expression to target cells will minimize off-target side effects, broadening the palette of deliverable therapeutics, and thereby improving safety and efficacy [8–10]. Recombinant adeno-associated virus (rAAV) is the gene-therapy vector of choice for many clinical applications, especially in non- or slowly-dividing cells, which are typical of the CNS [11–17]. However, the limited AAV-packaging capacity of ~4.9 kb [18] leaves very little space for a promoter. In contrast, mammalian promoters are generally large and complex, with cis-regulatory modules (CRMs) dispersed throughout the gene. Furthermore, identifying CRMs and predicting their function remains a major challenge [2, 19–21]. In this work, we take steps towards filling the research and expanding clinical need for small promoters with restricted expression by developing them for use in rAAV for the brain and eye.

We accomplished this goal by building upon our previously developed “MiniPromoters” (MiniPs), human DNA elements designed to drive expression in restricted cell types [22–25]. Previously, we bioinformatically parsed the entire human genome to identify genes suitable for MiniP design [24]. Further selection steps included genes with therapeutically interesting conserved expression patterns in human and mouse adult brain [26], and conserved computationally-predicted candidate regulatory regions (RRs). In general, MiniP designs were ~4 kb, consisting of a PROM (~1.1 kb at the transcription start site; range 254–3,536 bp) and multiple CRMs (~900 bp each; range 28–2,951 bp), which were usually non-contiguous and typically repositioned 5′ of the PROM. We named our MiniPs sequential by “Ple” number (for **Ple**iades Promoter Project). Initially, MiniPs were tested driving a reporter after being knocked-in, single-copy, site-specific 5′ of the mouse *Hprt* gene. Key features of this strategy included: 1) a large-scale pipeline, to reduce the impact of the expected high failure rate in promoter design; 2) physiologically relevant expression, potentially more suitable for therapeutic delivery; 3) human DNA, presumably decreasing our success when initially tested in mice, but favoring translation to humans if positive; and 4) the expectation that endogenous-like expression in mice best predicts a similar expression will occur in humans. In total, 45 such positive MiniPs have been developed [22, 24] and made available to the research community (www.addgene.org and www.jax.org).

However, we anticipate that not all MiniPs, which functioned as single-copy site-specific knock-ins (KIs) in the mouse genome, will also function specifically in rAAV. For example: 1) multiple virus copies may increase and thereby broaden expression; 2) developmentally-established epigenetic marks may not be acquired by a promoter introduced after birth; and 3) most of the original Pleiades promoters will need to be “cut down” to be useful in rAAV, which may inadvertently remove essential elements. However, we have previously tested three MiniPs developed in mice (one unaltered and two cut downs) in rAAV2 and were encouraged by their success [22]. On the other hand, this data set was small; thus, to draw a stronger conclusion and to provide more resources for the field, in this study we have evaluated a much larger set of 19 MiniPs. For this evaluation, we established a two-step, relatively large-scale high-throughput screening system for human MiniPs packaged in rAAV and tested in mouse. After promoter design, DNA was synthesized, cloned into our “plug and play” rAAV2 genome plasmid, packaged as rAAV2/9 for broad tropism, and injected intravenously into neonatal mice, with histological analysis of expression in adult brain and eye [27]. The first step of the screening uses a highly-sensitive historical-indirect reporter system, in which the MiniP drives icre (improved cre recombinase [28]) in a cre-reporter mouse. In the second screening step, representative successful promoters were recloned driving the direct reporter emerald green fluorescent protein (EmGFP) [29] and the assay performed again. By undertaking these studies with the smallest and thus most challenging virus, rAAV, we anticipate that successful promoters will have a wide use in other gene therapy modalities such as lentivirus, adenovirus, retrovirus, herpes simplex virus, plasmids, and nanoparticles.

## Results and Discussion

### Updated MiniPromoter virus pipeline allows for rapid construct screening

We had previously scored every gene in the human genome for suitability for MiniP design by assessing regulatory resolution, a metric that favors small genes with clearly defined conserved regions [24]. MiniPs were initially designed based on information in primary literature, transcription start sites, sequence boundaries, phylogenetic footprinting, and transcription factor binding site predictions [24]. Now, as additional genome-wide datasets such as RNAseq, and new data and tools within projects such as FANTOM [30] and JASPAR [31] become publically available, they were exploited for bioinformatically-driven MiniP design and redesign. In addition, compared to our previous work, the current pipeline was intended to move directly from MiniP design to a virus-based assay, decreasing the costs associated with KI mouse engineering, greatly reducing turn-around time, and making sequential redesigns feasible in the future.

**Fig. 1:**
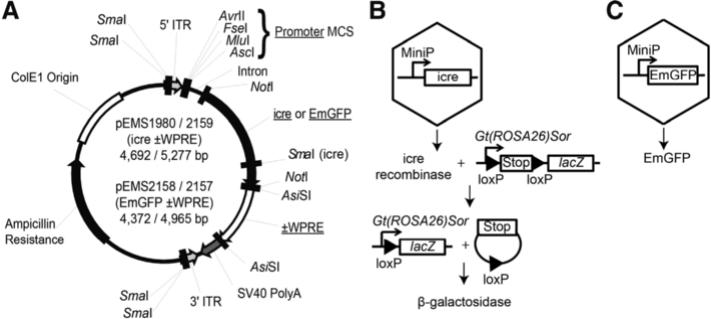
“Plug and Play” rAAV2 genome plasmid used to clone MiniPromoters (MiniPs) upstream of icre or EmGFP enables high throughput pipeline testing. **a** Plasmids were generated containing either the icre or the EmGFP reporter. An *Asi*SI site flanks a removable WPRE. MiniPs were cloned at the MCS using a combination of the four available cut sites. The plasmids are subsequently used to generate single stranded rAAV. **b** Screening step one, an historical-indirect reporter system. MiniPs drive expression of icre, which in turn recombines the endogenous loxP sites and removes the stop sequence 5ʹ of the *lacZ* gene, thus driving expression of β-galactosidase from the strong ubiquitous *ROSA26* promoter. **c** Screening step two, a direct reporter system. MiniPs drive direct expression of EmGFP, which can be imaged by epifluorescence or signal amplified using antibodies. bp, base pairs; ITR, inverted terminal repeat; MCS, multiple cloning site; WPRE, woodchuck hepatitis virus post-transcriptional regulatory element

In establishing this pipeline, we wanted a system that transduced as many cells as possible and showed expression with a ubiquitous promoter in all relevant cell types, such that any observed restriction would be attributable to the promoter. Thus, we tested 15 control rAAVs including: self-complementary and single-stranded genomes; AAV9 and AAVrh.10 serotypes; CMV, smCBA, CBA, CAGGS, and promoterless constructs; hGFP, GFP, cre, icre, and EmGFP reporters; and the 3′-UTR woodchuck hepatitis virus post-transcriptional regulatory element (WPRE). We used WPRE mut6, a DNA element known to substantially increase expression levels but without promoter activity [32, 33]. Figure 1a depicts the “plug and play” rAAV2 genome plasmid we developed, with AAV2-based inverted terminal repeats (ITRs), an intron, either icre or EmGFP reporters, with or without WPRE. Restriction enzyme sites allowed for the rapid exchange of promoters, reporters, or removal of the intron and/or WPRE. Figure 1b depicts the first evaluation step of the pipeline, in which a MiniP drove icre, which permanently removed the “stop” from a mouse genomic locus where the ubiquitous *ROSA26* promoter drove a “lox-stop-lox-*lacZ*” allele, resulting in high-level expression of *lacZ* wherever and whenever icre was expressed. Thus, this step used an historical-indirect reporter of cre activity. Figure 1c depicts the second evaluation step of the pipeline, in which a positive MiniP was retested driving EmGFP, a direct reporter of MiniP expression, detected via epifluorescence or immunofluorescence staining.

### MiniPromoter selection surveyed a variety of design types

**Fig. 2:**
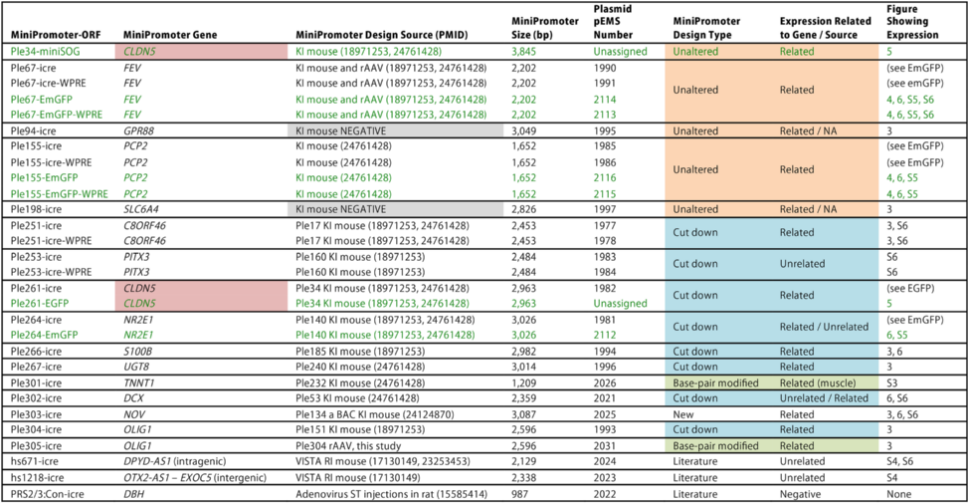
Twenty-nine rAAVs, carrying 19 MiniPromoters (MiniPs), from 17 gene/loci, resulted in 16 MiniPs with expression patterns related to the gene/source. MiniPs in black text drive icre; MiniPs in green text drive a green fluorescent protein; horizontal lines group MiniP constructs; note that the *CLDN5*-based MiniPs are separated in the table and thus highlighted in pink; BAC, bacterial artificial chromosome; bp, base pairs; EmGFP; emerald green fluorescent protein; icre, improved cre recombinase; KI, knock-in; miniSOG, mini Singlet Oxygen Generator; NA, not applicable; ORF, open reading frame; PMID, PubMed ID number; rAAV; recombinant adeno associated virus; ST, stereotaxic; VISTA RI, random insertion from VISTA enhancer project; WPRE, woodchuck hepatitis virus post-transcriptional regulatory element

Figure 2 lists the 29 experimental viruses generated for this project. The 19 MiniPs carried by these viruses were all chosen for potential therapeutic utility. The majority of our work consisted of P0 virus injections for both icre and EmGFP viruses. A subset of EmGFP constructs were also injected at P4, since it has been shown that the developmental age at which virus is delivered to the mouse eye determines the cell types most efficiently transduced [34].

Five previously designed MiniPs were tested “unaltered” in rAAV2/9: Ple34 (*CLDN5* RRs), Ple67 (*FEV* RRs), Ple94 (*GPR88* RRs), Ple155 (*PCP2* RRs), and Ple198 (*SLC6A4* RRs) [22, 24]. KI mouse data for three of these MiniPs (Ple34, Ple67, and Ple155) had been previously published [22, 24], of which Ple67 was also known to drive expression in the eye following intravitreal rAAV delivery [22]. The remaining two MiniPs in this group (Ple94 and Ple198), were chosen despite negative KI mouse data due to their potential clinical impact, as we suspected detection was a limitation with a subset of our previously negative single-copy EGFP reporter KI mice [24]. Our hypothesis was that the multi-copy rAAV system would enhance low-level expression, allowing us to recapture previously “negative” MiniPs.

A further eight previously designed MiniPs, which were positive in KI mice, were chosen for “cut down” to ~2.5 kb or smaller, after bioinformatics reanalysis identified regions that were less likely to contribute to regulatory function (Ple251 (*C8ORF46* RRs), Ple253 (*PITX3* RRs), Ple261 (*CLDN5* RRs), Ple264 (*NR2E1* RRs), Ple266 (*S100B* RRs), Ple267 (*UGT8* RRs), Ple302 (*DCX* RRs), and Ple304 (*OLIG1* RRs)). The majority of the original Pleiades MiniPs require such modification in order to be useful in the space-limited rAAV as they were initially ~4 kb for KI mouse studies.

For two MiniPs (Ple301 (*TNNT1* RRs) and Ple305 (*OLIG1* RRs)) we undertook bioinformatically-driven “base-pair modifications” that were divergent from the reference human sequence, in favor of a transcription factor consensus sequence designed to strengthen the previously successful promoter. Thus, we included in the study Ple301, which carried two base-pair modifications compared to our previous Ple232, which was positive in KI mice [22]. The base-pair modifications were introduced to improve the core promoter region containing downstream core elements (DCEs), which are recognized by TFIID [35]. Similarly, Ple305 was a three base-pair modification of Ple304 (a cut-down described above). The changes were designed to increase expression by strengthening the TATA-box and upstream B recognition elements (BREs) [36, 37].

Three promoters were also chosen from the “literature”. Two were representative of the extensive community resource developed by the VISTA enhancer project, the main goal of which is to identify distant-acting non-coding regulatory elements that drive developmental gene expression in a region-specific manner [38]. VISTA enhancers are chosen using extreme evolutionary sequence conservation and/or ChIP-seq data, and then tested combined with a mouse minimal promoter (*Hsp68*) by random insertion in the mouse genome, with expression evaluated in E11.5 embryos. In our system, we tested two of their small human enhancers that had highly-specific brain expression: hs671 (forebrain; *DPYD-AS1* intragenic genomic location), and hs1218 (midbrain; *OTX2-AS1 – EXOC5* intergenic genomic location). A third literature example was chosen due to its important therapeutic application in Parkinson disease. Although, in our previous work we were able to obtain regionalized expression with a *DBH*-based MiniP in KI mice, there was no clear overlap with tyrosine hydroxylase staining [24]. Thus, we choose to test PRS2/3:Con; a *DBH*-based promoter that had been successfully used in adenovirus in the rat adult brain [39].

Lastly, we computationally designed one completely new MiniP for direct testing in rAAV to assess our *de novo* design abilities. For this work, we chose *NOV* (nephroblastoma overexpressed gene). Previously, because *NOV* had a poor RR score of 0.0439 primarily due to a large genomic region [24], despite clear TSS/promoter and RR elements, we had tested a retrofitted *NOV* BAC in a *Hprt* KI mouse with promising positive results [2]. Although a BAC construct may not delineate specific regulatory elements, it does provide genomic boundaries for the regulatory region. Thus, we designed a MiniP using elements within the *NOV* BAC, for testing in virus.

### Ubiquitous promoters demonstrate widespread transduction

After an initial wide survey of methodologies, we focused on neo-natal intravenous delivery of single-stranded rAAV in AAV9, due to its excellent capability to transduce a variety of CNS cell types in our hands and as described in the literature [27, 40, 41]. To evaluate the reproducibility of these methods the expression of icre from the CAGGS and EmGFP from the CAGGS and smCBA ubiquitous promoters was analyzed. Consistent staining was seen, with positive staining in all brain regions, but reduced in areas such as the striatum, midbrain, ventral hindbrain, and Purkinje cells of the cerebellum (Additional file 1: Figure S1). With the direct EmGFP reporter, expression was markedly increased in intensity and in number of positive cells when combined with the WPRE.

These results are consistent with the literature on ubiquitous promoters, which indicates variation in their cell-type specific expression. The cell-type variation in ubiquitous promoter expression may result in inadequate reporting on the full spectrum of viral tropism for a particular AAV and delivery method [42–44]. For instance, the observation of poor expression in motor neurons with the CBA ubiquitous promoter, only to find strong expression when using a MeCP2-based promoter, which has neuronal expression in the CNS [43]. Thus, we propose that the true breadth of viral tropism in our system will be determined by the overlap of ubiquitous and specific promoters.

### 16 novel MiniPromoters with consistent restricted expression

In total, 19 Pleiades MiniPs were tested in rAAV2/9. Eighteen were tested driving icre in mice carrying a “lox-stop-lox-*lacZ*” allele, an historical-indirect reporter. Four of these were further tested driving either EGFP or EmGFP direct expression reporters. To avoid duplication, we only present the GFP data for these four. Finally, one Pleaides MiniP was tested driving miniSOG, a direct expression reporter. In all, we saw 16 MiniPs with restricted expression: 11 in the brain, 10 in the eye, and one in muscle.

The consistency of MiniPromoter constructs was assessed by analyzing expression patterns in multiple animals (Additional file 1: Figure S2). In the brain, all 19 MiniPromoter constructs showed ≥75 % consistency with the figures presented (*n* ≥ 4 animals per construct). In the eye, 11/13 MiniPromoter constructs showed ≥70 % consistency with the figures presented (*n* ≥ 4 animals per construct).

### MiniPromoters for the brain include restricted expression in Raphe nuclei and blood brain barrier

**Fig. 3:**
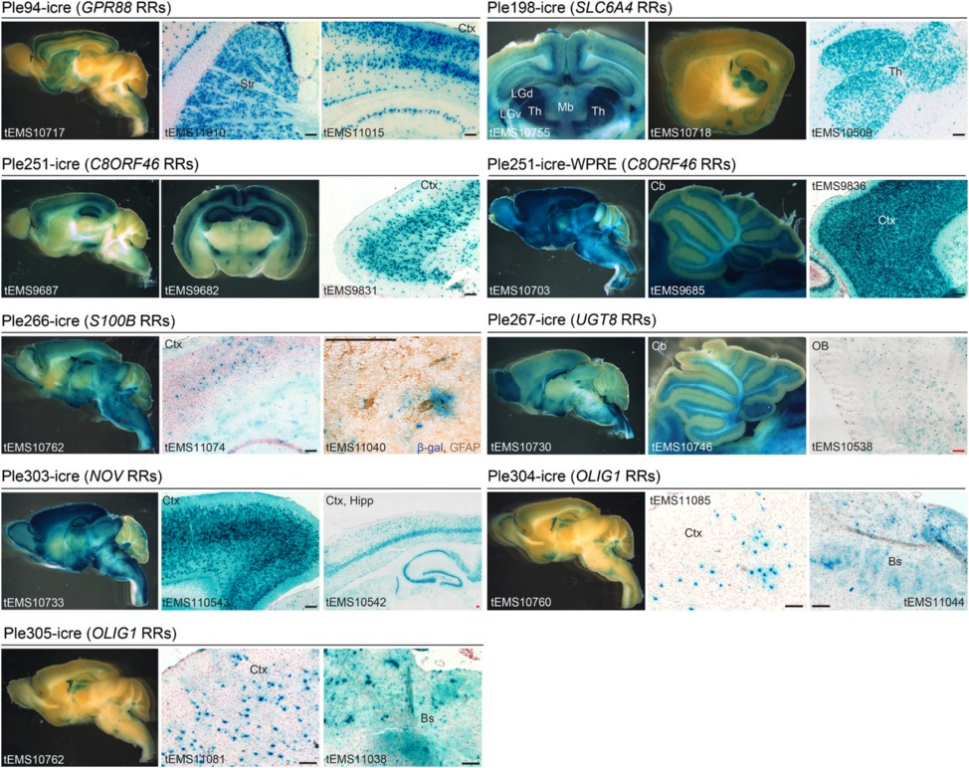
Eight MiniPromoters in rAAVs show unique expression related to their source gene when driving icre in mice carrying a “lox-stop-lox-*lacZ*” allele, an historical-indirect reporter. Each tEMS number indicates an individual animal. Ple94-icre (*GPR88* RRs) expressed strongly in the striatum and in upper cortical layers. Ple198-icre (*SLC6A4* RRs) expressed in the thalamus and shows a clear demarcation between LGd and LGv. Ple251-icre (*C8ORF46* RRs) demonstrated strong expression in the hippocampus and cortex; layers IV, V, and Vb are predominant. The zona incerta is also labeled. Ple251-icre-WPRE (*C8ORF48* RRs) extended the expression to most brain regions, particularly throughout the cortex and in the cerebellar granular layer. Ple266-icre (*S100B* RRs) expressed sporadically in the brain with puffy processes in the cortex and corpus callosum. As expected for partial viral transduction, a subset of GFAP+ astrocytes are co-labelled with β-gal. Ple267-icre (*UGT8* RRs) has strong staining in the olfactory bulb and in the cerebellar granule layer, with some Purkinje cells labeled. In the OB, globeruli-like structures are stained. Ple303-icre (*NOV* RRs) stains all cortical layers in midline and becomes specific to cortical layer V in more lateral sections. Ple304-icre and Ple305-icre (both *OLIG1* RRs) demonstrated an indistinguishable expression pattern with scattered cells in several brain regions, including the cortex and brainstem, resembling puffy processes of oligodendrocytes. Bs, brainstem; Cb, cerebellum; Ctx, cortex; Hipp, hippocampus; icre, improved cre recombinase; LGd, dorsal lateral geniculate; LGv, ventral lateral geniculate; Mb, midbrain; OB, olfactory bulb; RRs, regulatory regions; Str, striatum; Th, thalamus. Blue, β-gal positive; brown, DAB immunohistochemistry; red, neutral red. [Scale bars = 100 μm]

Figure 3 presents the data for eight positive Pleiades MiniPs driving icre. We chose to first test MiniPs with the historical-indirect reporter system because of its high sensitivity, since any expression of icre, from the neonatal introduction of the virus to the adult harvest, would be captured by the strong genomic *Gt(ROSA)26Sor* promoter. Nevertheless, we were surprised by the generalized pattern of positive staining that was sparse, but widespread, and similar across all well-injected mice. This “background” may have been due to non-specific icre expression as a result of initial high viral copy number, the time required for appropriate epigenetic marks, and transcription factor binding. However, superimposed on this was the expected restricted expression for many of the MiniPs, suggesting this problem resolves. For example, two previously negative KI designs, Ple94 (*GPR88* RRs) and Ple198 (*SLC6A4* RRs), were positive and expressed in the striatum and thalamus respectively – as expected by the endogenous gene expression in development and/or the adult mouse [45–49]. We hypothesize that the KI mice may have expressed below detection levels either due to being single-copy, EGFP, or both. Notice also that the Ple94 MiniPromoter showed strong expression in the striatum, a region of weak expression with the ubiquitous promoter, thus expanding the characterization of the viral tropism in this screening system (Additional file 1: Figure S1). We also examined the effect of the WPRE on expression specificity, and noticed a very strong increase in the number of positive cells with the Ple251 (*C8ORF46* RRs) MiniP. While Ple251-icre expressed in limited cortical layers, the hippocampus, and the zona incerta; the addition of the WPRE generalized expression to a near ubiquitous pattern. This result suggests that in this system, WPRE greatly enhances low-level and undetectable transcript levels. In addition, the current set includes four constructs that expressed in putative glial cells (Ple266 (*S100B* RRs), Ple267 (*UGT8* RRs), Ple304 (*OLIG1* RRs), and Ple305 (*OLIG1* RRs)). Ple304 and Ple305 exhibited highly similar expression patterns, despite changes to the latter designed to strengthen expression. Finally, using our Ple303 MiniP, we demonstrate similar expression to the previous BAC construct [2], indicating success with our *de novo* design strategy.

Of course, negative, or partially negative results were also obtained. First, minor base-pair modifications were made to our previous Ple232 (*TNNT1* RRs) MiniP, resulting in Ple301. In doing so, surprisingly, we lost the zona incerta brain expression seen previously in Ple232 KI mice [22], yet retained muscle specificity (Additional file 1: Figure S3). This serves as an example where moving the promoter to virus and/or minor base-pair modification altered the function of the promoter in one cell type, but not another. Second, when we tested hs671 and hs1218 each linked to the *Hsp68* mouse basal promoter as they were in the VISTA Enhancer project [38], we did not observe the expected regional specificity to either the forebrain or midbrain respectively (Additional file 1: Figure S4). Expression was noted in those regions, but similar expression was also detected throughout most other brain regions. Thus, we deemed these two constructs as being non-specific in our system. We hypothesize that these VISTA enhancers may only restrict expression embryonically, and not postnatally. We also tested from the literature PRS2/3:Con, a *DBH*-based promoter that was successfully used in adenovirus in the rat brain [39]. However, we found that with intravenous P0 delivery using rAAV it showed no expression different from background. We hypothesize that this difference may have been due to the developmental stage of delivery (P0 versus adult), the virus utilized, or the use of human sequence in mouse versus rat.

**Fig. 4:**
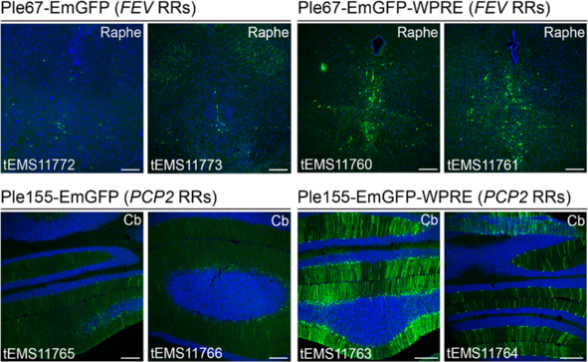
Two MiniPromoters selected based on icre expression, recapitulated their unique expression related to their source gene when tested driving EmGFP. Ple67-EmGFP ± WPRE (*FEV* RRs) drove expression in the dorsal raphe nuclei of the mouse brain. Ple155-EmGFP ± WPRE (*PCP2* RRs) drove expression in the Purkinje cells of the cerebellum, similar to the endogenous gene. In both cases, the presence of WPRE enhanced the number of cells and intensity of cells showing expression. Cb, cerebellum; EmGFP, emerald green fluorescent protein; Raphe, raphe nuclei; RRs, regulatory regions; WPRE, woodchuck hepatitis virus post-transcriptional regulatory element. Blue, Hoechst 33342; Green, EmGFP immunofluorescence. [Scale bars = 200 μm]

In the second screening step, representative successful promoters from step one were recloned driving the direct reporter EmGFP. Figure 4 demonstrates that the Ple67 (*FEV* RRs) and Ple155 (*PCP2* RRs) MiniPs drive highly specific and expected expression when injected at P4 in the temporal vein. Additional file 1: Figure S5 presents additional supportive data for both these MiniPs injected at P0. Ple67 was derived from *FEV*, a gene expressed in the serotonergic neurons of the raphe nuclei in the brain [50]. Previously, Ple67-EGFP and -*lacZ* KI mice gave the expected raphe expression [22, 24]. Here, using rAAV, Ple67 again localized expression to the raphe, which was further enhanced with the WPRE (Fig. 4 and Additional file 1: Figure S5). Ple155 was derived from *PCP2,* a gene expressed in the Purkinje cells of the cerebellum [51, 52]. However, Ple155-*lacZ* KI mice did not show this expected expression [22]. Thus, it was surprising when using rAAV, Ple155 gave strong and consistent expression in the Purkinje cells and their processes, which was further enhanced with the WPRE (Fig. 4, Additional file 1: Figure S5). Since expression with the ubiquitous promoters, even when enhanced with WPRE, was limited in Purkinje cells (Additional file 1: Figure S1), this result further expands the characterization of the viral tropism in this system. This result also suggests that KI at the *Hprt* genomic location is not permissive for *PCP2*-based expression in Purkinje cells, or that the presumed multi-copy expression of virus in Purkinje cells may push the reporter above detection levels compared to the KI mouse. As anticipated, negative MiniP results were also obtained. Additional file 1: Figure S5 shows Ple264, which lacks elements 12 and 13 compared to the original Ple140 (*NR2E1* RRs). While Ple140 was strongly positive in the hypothalamus of adult and embryonic KI mice [22, 24], Ple264 lost the hypothalamic expression when delivered in rAAV. This was presumably attributable to either the “cut down” or the viral delivery system.

**Fig. 5:**
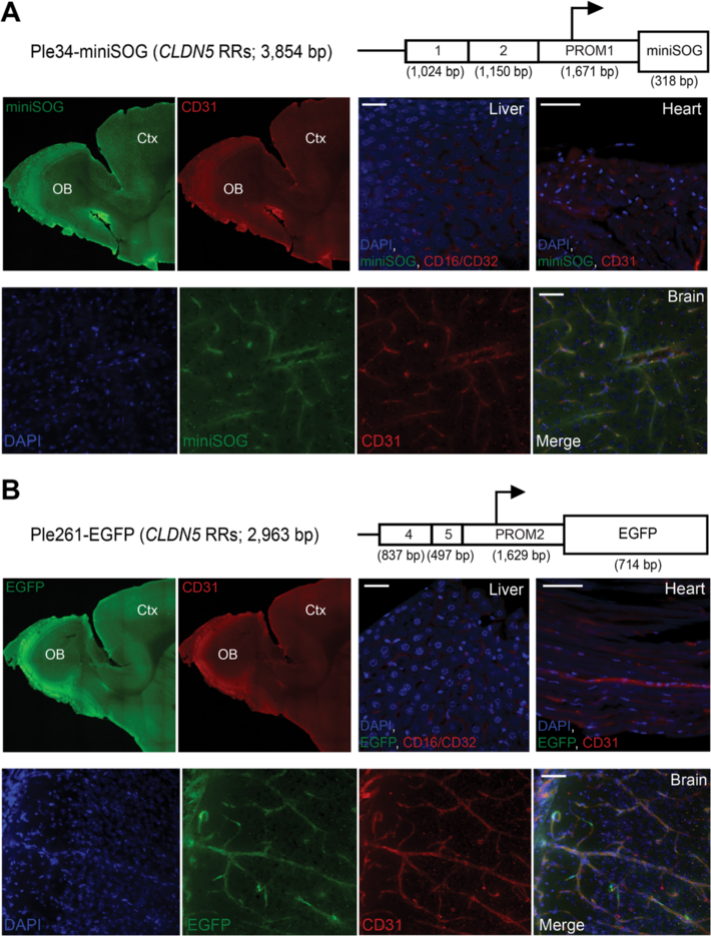
Two *CLDN5* RRs-based MiniPromoters (MiniPs) showed brain endothelial cell-specific expression related to their source gene, when virus was introduced into the adult circulatory system. **a** The Ple34 MiniP drove expression of the miniSOG fluorescent green reporter in blood vessels in the brain. Co-labelling was observed with CD31, a marker of endothelial cells. No miniSOG expression was detected in the heart where CD31 stains endocardial endothelium, or liver, where CD16/32 stains endothelial cells. **b** Ple261, a “cut down” of Ple34, also drove expression of EGFP in blood vessels in the brain. Again, co-labelling was observed with CD31, a marker of endothelial cells, but no expression was detected in the heart or liver. bp, base pairs; Ctx, cortex; EGFP, enhanced green fluorescent protein; miniSOG, mini Singlet Oxygen Generator; OB, olfactory bulb; RRs, regulatory regions. [Scale bars = 50 μm]

Figure 5 presents two examples where the MiniP has been taken beyond screening, to modeling delivery applicable to gene therapy. For this, we evaluated the ability of Ple34 and Ple261 (*CLDN5* RRs) to drive expression in the endothelial cells of the blood brain barrier, an important therapeutic target. Ple34 was previously positive driving *lacZ* in a KI mouse [24], and the “cut down” version, Ple261 (Fig. 2), was tested driving icre by temporal vein injection at P0. However, tail vein injection in adult mice models a possible therapeutic delivery, and allows evaluation of the expression with a mature blood-brain barrier. Figure 5a and b respectively, show consistent overlap of Ple34-miniSOG (a fluorescent reporter chosen for its small size [53]) and Ple261-EGFP, with the endothelial cell marker CD31. Importantly, MiniP-driven miniSOG or EGFP co-labeling with endothelial markers in the liver (CD16/CD32) and heart (CD31) was absent.

Overall, this data demonstrates that many of the Pleiades MiniPs expressed in rAAV according to their design source and thus may be useful for rAAV-mediated “gene-of-interest” delivery in the brain.

### MiniPromoters for the eye include restricted expression in bipolar ON cells and Müller glia

Due to developmental similarities, we anticipated that many of the MiniPs designed for use in the brain would also be applicable to basic, preclinical, and even clinical use in ocular gene therapy. Thus, all 19 Pleiades MiniPs tested in rAAV2/9 were examined for expression in the eye after intravenous injection into neonatal mice. As in the brain, the historical-indirect icre reporter system gave a generalized pattern of positive staining that was sparse but widespread. However, in some cases superimposed on this was the expected restricted expression. As with the brain, only the GFP data is presented for MiniPs selected for second-step screening to avoid duplication. In all, we obtained restricted expression in 10 MiniPs in the eye.

**Fig. 6:**
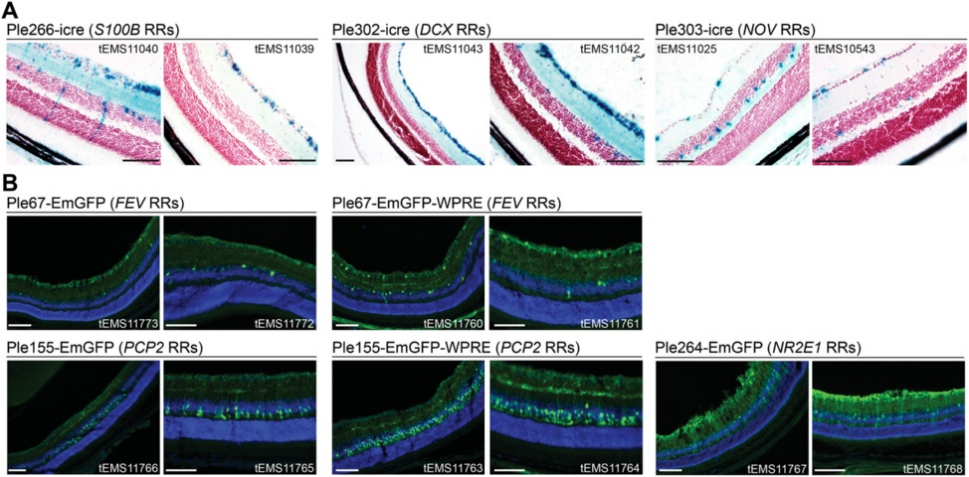
Six MiniPromoters (MiniPs) showed cell type-specific expression in the retina. **a** The icre reporter led to identification of three retina positive MiniPs. Ple266-icre (*S100B* RRs) showed rare Müller glia expression as expected for the endogenous gene in one of two mice tested [69]. The astrocytic layer also demonstrated some staining. Ple302-icre (*DCX* RRs) intensely stained the ganglion cell layer (GCL), similar to previous *DCX*-based MiniPs [22]. For Ple303-icre (*NOV* RRs) we observed modest enrichment of staining in the outer side of the inner nuclear layer indicative of horizontal cells. Blue, β-gal positive; red, neutral red. **b** Constructs utilizing the EmGFP reporter identified an additional three MiniPs with retinal expression. In both cases tested, the presence of the WPRE enhanced the number and intensity of cells showing expression. Ple67-EmGFP ± WPRE (*FEV* RRs) demonstrated expression primarily in the GCL and inner nuclear layer in putative ganglion and amacrine cells. Rarely, additional cells were also positive with the WPRE-containing virus. Ple155-EmGFP ± WPRE (*PCP2* RRs) restricted expression to bipolar ON cells. Ple264-EmGFP (*NR2E1* RRs) showed strong staining of Müller glial cells. EmGFP, emerald green fluorescent protein; GCL, retinal ganglion cell layer; icre, improved cre recombinase; RRs, regulatory regions. [Scale bars = 100 μm]

Figure 6a presents the data for three positive Pleiades MiniPs driving the historical-indirect reporter icre in the retina of “lox-stop-lox-*lacZ*” mice after injection at P0. Ple266 (*S100B* RRs) stained rare Müller glia (one of two mice) above the background. Ple302 (*DCX* RRs) intensely stained the retinal ganglion cell layer (GCL). Ple302 is a “cut down” of Ple53 (Fig. 2), which in a KI mouse showed expression in the GCL, but also neurogenic regions of the brain [22, 24]. However, when Ple302 was used in rAAV, the restricted brain expression was lost, either due to the “cut down” or the viral delivery system. Notable is that the GCL is not a region of endogenous *DCX* expression, but has been previously observed with another promoter similar to Ple53 [54]. Finally, Ple303 (*NOV* RRs) showed some modest enrichment in horizontal cells.

In the second screening step, MiniPs driving the direct reporter EmGFP were positive in the retina and cornea after injection at P4. In Fig. 6b retinal expression is presented. Ple67 (*FEV* RRs), showed expression primarily in the GCL and inner nuclear layer, indicative of putative ganglion and amacrine cells, which was enhanced by the presence of the WPRE. Ple155 (*PCP2* RRs) showed restricted expression in bipolar ON cells. In this case, the presence of the WPRE allowed enhanced visualization of the cellular end feet. Note, comparison with previous work demonstrates that the expression of Ple67 and Ple155 is serotype and delivery-method independent [22, 55]. Finally, Ple264 (*NR2E1* RRs) gave strong Müller glia staining, another gene therapy target [56]. Interestingly, Ple264 is a “cut down” of Ple140 which in KI mice expressed strongly in the hypothalamus, but was completely negative in the eye [22]. In Ple264, after the removal of elements 12 and 13, the brain expression was lost, either due to the “cut down” or viral delivery. However, Ple264 gained the *NR2E1*-endogenous expression in Müller glia [57, 58], either due to removal of inhibitory sequences from the promoter, the multi-copy rAAV system pushing the reporter above detection levels compared to the KI mouse, or non-permissive expression conditions at the *Hprt* genomic location for *NR2E1*-based expression in the KI mouse.

In the cornea (Additional file 1: Figure S6), we observed expression for five MiniPs driving icre (Ple251 (*C8ORF46* RRs), Ple253 (*PITX3* RR), hs671, Ple302 (*DCX* RRs), and Ple303 (*NOV* RRs), and one driving EmGFP (Ple67 (*FEV* RRs)). Typically, expression was stromal, but Ple251 also showed expression in the epithelial layer of the cornea. In the case of Ple251-icre, Ple253-icre, and Ple67-EmGFP, we were unable to detect corneal expression without the use of the WPRE.

Overall, this data represents a rich resource of therapeutically-enabling eye MiniPs that are likely to have efficacy similar to the preclinical use of the Ple155 MiniP, which has already been used to restore vision in a mouse model of congenital night blindness [55].

We have demonstrated that a single-step rAAV pipeline utilizing a direct reporter is sufficient for MiniP development. We initially established a two-step screening system, assuming we would need the sensitivity of a historical-indirect reporter system to detect expression from restricted promoters. However, we have found there are disadvantages to such a sensitive system, and that detection of the direct EmGFP reporter was not limiting, perhaps because of the multi-copy rAAV system, although occasionally requiring anti-GFP immunohistochemistry or a WPRE for increased expression. Importantly for this approach, we showed that the positive impact of WPRE was independent of MiniP, or open reading frame, and that in the two cases for which cell specificity could be best determined (Ple67-EmGFP and Ple155-EmGFP (Figs. 4, 6)), expression in the brain and retina was not “broadened” by WPRE. Given this data, we suggest utilization of a single-step direct reporter and the WPRE in future work.

The 16 MiniPs (human DNA elements designed to drive expression in restricted cell types) presented here are important advances for basic and preclinical research, and may enable a paradigm shift in clinical gene therapy. To assess the effectiveness of moving MiniPs from KI mice to rAAV, we utilized five previous Pleiades designs and 100 % showed expression related to the gene and source. This demonstrates that the previously published Pleiades MiniPs provide a rich resource of constructs for use in rAAV. However, many previous MiniPs will still need to be “cut-down” for use in rAAV. Thus, we undertook this work for eight Pleiades MiniPs, of which five demonstrated expression related to their gene and source, and two MiniPs maintained a further restricted aspect of their previous expression. Therefore, 87.5 % maintained similar expression patterns despite extensive size reduction. In addition, for one gene we attempted, and successfully developed, a new promoter rapidly and inexpensively. Finally, we demonstrate the ability to test MiniPs directly in a single step in the mouse EmGFP viral pipeline. Therefore, we provide extensive evidence for the utilization of a high-throughput method of promoter design and testing in rAAV. These MiniPs are now available unrestricted to the academic community.

## Methods

### MiniPromoter bioinformatics and design

MiniPromoter (MiniP) bioinformatics has been described in part, previously [24]. For our cut-downs and new designs we considered the UCSC genome browser [59] tracks: ENCODE datasets [60–66], TFBS conserved, VISTA enhancers [38], UCSF brain methylation, ORegAnno [67, 68], CpG islands, human mRNAs/ESTs, CD34 DNAse I, Repeat Masker, and Conservation. For the Conservation tracks, we utilized the mammalian conservation and Multiz alignments including placental mammals and vertebrates, but excluded non-human primates to minimize selection bias that may be introduced by high sequence similarities between humans and primates. The following histone modifications were considered: methylation of H3K4 and H3K36 as indicators of transcriptional activation, methylation of H3K9 and H3K27 for transcriptional silencing, and acetylation of H3K4 and H3K27 indicating transcriptional activation. Tracks such as TFBS conserved, VISTA enhancers, and ORegAnno provide *in silico* and/or experimental evidence for transcription factor binding sites and regulatory regions. Tracks for DNAse I hypersensitivity, such as CD34 DNAse I, help to mark regions that tend to be associated with open chromatin structure and may contain active binding sites. Potential regulatory regions were rated as containing 1 to 5+ features; the highest scores typically being chosen for testing in MiniPs.

### Mice

We purchased heterozygous 129S-*Gt(ROSA)26Sor*^*tm1Sor*^/J mice at N? + 9p (JAX Stock No: 003310) and continued backcrossing the allele onto the 129S1/SvImJ strain (JAX Stock No: 002448) until N13. Heterozygous mice were then intercrossed to obtain a congenic homozygous strain. We also purchased homozygous B6.129S4-*Gt(ROSA)26Sor*^*tm1Sor*^/J mice at N4F10N1p (JAX Stock No: 003474) and restarted backcrossing the allele onto the C57BL/6J strain (JAX Stock No: 000664) until N11. Heterozygous mice were then intercrossed to obtain a congenic homozygous strain. All experimental mice were B6129F1 hybrids and generated either as the first generation of crossing a congenic homozygous 129S-*Gt(ROSA)26Sor*^*tm1Sor*^/JEms to a congenic homozygous B6.129S4-*Gt(ROSA)26Sor*^*tm1Sor*^/JEms, or C57BL/6J to 129S1/SvImJ. For clarity, we describe mice carrying the *Gt(ROSA)25Sor*^*tm1Sor*^ allele as containing a “lox-stop-lox-*lacZ*” reporter, which we utilized as an indirect-historical marker of cre-recombinase expression.

### Virus generation and analysis

#### Virus production

We generated a “plug and play” rAAV2 genome plasmid that included AAV2 inverted terminal repeats (ITR), and allowed for the easy exchange of promoters, intron, reporters, and 3’ UTR elements using restriction enzymes. In this study we tested the 3’ UTR element WPRE (woodchuck hepatitis virus post-transcriptional regulatory element) mut6 [32, 33]. MiniPs were isolated from existing Pleiades Promoter plasmids [22, 24], or generated by direct synthesis (DNA2.0, Menlo Park, CA, USA), and cloned into the multiple-cloning site (MCS) of a new “plug and play” rAAV2 genome plasmid - either the pEMS1987 backbone containing the improved cre (icre) reporter [28], and/or the pEMS2157 backbone containing the emerald green fluorescent protein (EmGFP) reporter [29]. Plasmids were propagated in the *E. coli* SURE Cells (Agilent Technologies, Santa Clara, CA, USA). One to five μg of plasmid DNA containing the MiniP was prepared by QIAgen Spin MiniPrep Kit (Catalog #27104, QIAgen, Germantown, MD, USA). rAAV plasmid DNAs was demonstrated free of rearrangements by *Ahd*I digest, ITRs verified via *Sma*I single digests, and overall structure via *Asc*I/*Eco*RI double digestion, and upon confirmation, sent to the Vector Core at the University of Pennsylvania (Philadelphia, PA, USA) for large-scale DNA amplification using the EndoFree Plasmid Mega Kit (Catalog #21381, QIAgen, Germantown, MD, USA). Quality control on the plasmid preparation was done via *Sma*I, *Pvu*II, and *Sna*BI confirmatory digests, and subsequently packaged into rAAV2/9 serotype virus. Ple34-miniSOG and Ple261-EGFP were cloned, DNA amplified, and packaged into AAV2/9 in the laboratory of A.A. for adult intravenous experiments.

#### Virus injection

B6129F1 hybrid pups were either homozygous for the *Gt(ROSA)26So*r^*tm1Sor*^ allele for icre virus injections, or wild type at that locus for EmGFP virus injections. Timed pregnancies were achieved using crowded females, experienced studs, and plug checking of females such that the day of birth could be accurately predicted. Virus injections were into postnatal (P) 0 (the day of birth) pups for icre constructs, and P4 for EmGFP constructs. For P0, if the female gave birth over night or in the morning, virus was injected in the afternoon. If she gave birth in the afternoon, virus was injected the next morning. Standard P0 and P4 pup injections were into the superficial temporal vein using 1 × 10^13^ GC/mL (genome copies per milliliter) virus in a volume of 50 μL (in PBS) with a 30-gauge needle and a 1 cc syringe. After injection, pups were tattooed for identification and returned to their cage. Ple34-miniSOG (dose of 3 × 10^11^ viral genomes) and Ple261-EGFP (dose of 8 × 10^11^ viral genomes) virus were injected intravenously at 6–8 weeks via tail vein.

#### Harvesting of animals

Virus-injected mice were harvested at P21 or P56. Animals were given a lethal dose of avertin injected intraperitoneally. Thereafter, perfusion with 1xPBS for 2 min and 4 % PFA/PBS for 8 min was performed. Brain, eye, spinal cord, and heart were harvested and post-fixed for 2 h at 4 °C. The tissues were then stored in 0.01 % azide/PBS at 4 °C. Ple34-miniSOG and Ple261-EGFP mice were harvested 4 weeks post injection.

#### Histology

Reporter gene expression was usually analyzed at P21 or P56 in brain, eye, spinal cord, and heart. Tissues were cryoprotected in 25 % sucrose/PBS overnight at 4 °C. After embedment in OCT (optimal cutting temperature compound) the following day, 20 μm sections were directly mounted onto slides. For X-gal (5-bromo-4-chloro-3-indolyl-β-D-galacto-pyranoside) staining, tissues were rinsed in PBS and Triton-X/PBS and stained in 0.1 % X-gal solution overnight (~18 h) at 37 °C. After staining, sections were rinsed and counterstained with neutral red, dehydrated, and mounted with coverslips. For co-labeling of X-gal with markers using immunohistochemistry, standard procedures were followed and the X-gal stain was performed either prior to primary antibody incubation or between primary and secondary antibodies, depending on the strength of the X-gal stain. X-gal stains blue any cells that have recombined the “lox-stop-lox-*lacZ*” reporter locus due to icre recombinase activity and thus expressing the β-galactosidase protein. With the icre reporter, we detected historical and indirect expression of the promoter.

EmGFP constructs were signal amplified using a chicken α-GFP antibody (Aves Labs Inc.; 1:500) and Alexa Fluor 488 secondary (Life Technologies; 1:1000). With the EmGFP fluorescent reporter, we visualized direct expression. Ple34-miniSOG and Ple261-EGFP mice were analyzed for green epifluorescence (miniSOG or EGFP) and co-stained with markers of endothelial cells (brain and heart: CD31; liver: CD16/CD32).

### Imaging

Between four and twelve animals were studied for each MiniPromoter, and unique expression patterns of the MiniPs were determined by microscope and image analyses. The images shown are typically from mice that had the most successful injections and thus the full dose of rAAV, and therefore the greatest likelihood to observe off-target expression if present. Brightfield and fluorescence images were standardly taken using an Olympus BX61 motorized microscope or a Zeiss 710 Confocal Laser Scanning Microscope. Images were processed using ImageJ (http://rsbweb.nih.gov/ij/), Photoshop, and Illustrator (Adobe, San Jose, CA). Brightness, contrast, and color balancing adjustments, as per Molecular Brain guidelines, were made where necessary to improve visibility.

### Statement of ethics approval

All procedures involving animals were in accordance with the Canadian Council on Animal Care and University of British Columbia Animal Care Committee (protocols #A10-0268, and A10-0269). All animal experiments were carried out in accordance to NIH guidelines and as approved by the UNC Institutional Animal Care and Use Committee (IACUC).

### Statement of consent for publication

Not applicable.

### Materials and data availability

All MiniPs and constructs have been deposited with AddGene (www.addgene.org), with the exception of those constructs used in generating Fig. 5 – these are available from A.A. upon request (aravind_asokan@med.unc.edu).

## Additional file

Additional file 1: Supplementary Figures 1 - 6. (PDF 10 kb)

## Abbreviations

BAC: bacterial artificial chromosome
bp: base pairs
Bs: brainstem
Cb: cerebellum
Ctx: cortex
EGFP: enhanced green fluorescent protein
EmGFP: emerald green fluorescent protein
GCL: retinal ganglion cell layer
Hipp: hippocampus
icre: improved cre recombinase
ITR: inverted terminal repeat
KI: knock-in
LGd: dorsal lateral geniculate
LGv: ventral lateral geniculate
Mb: midbrain
MCS: multiple cloning site
MiniP: MiniPromoter
miniSOG: mini Singlet Oxygen Generator
NA: not applicable
OB: olfactory bulb
ORF: open reading frame
PMID: PubMed ID number
rAAV: recombinant adeno associated virus
Raphe: raphe nuclei
RRs: regulatory regions
ST: stereotaxic
Str: striatum
Th: thalamus
VISTA RI: random insertion from VISTA enhancer project
WPRE: woodchuck hepatitis virus post-transcriptional regulatory element
