# Supplementary material for: rAAV-compatible MiniPromoters for restricted expression in the brain and eye
Source: Mol Brain. 2016 May 10;9:52. doi: 10.1186/s13041-016-0232-4 (PMC4862195; doi:10.1186/s13041-016-0232-4)
Supplement: Additional file 1: — Supplementary Figures 1 - 6. (PDF 10 kb) [file 13041_2016_232_MOESM1_ESM.pdf]

# rAAV-Compatible MiniPromoters for Restricted Expression in the Brain and Eye

Charles N. de Leeuw, Andrea J. Korecki, Garrett E. Berry, Jack W. Hickmott, Siu Ling Lam, Tess C. Lengyell, Russell J. Bonaguro, Lisa J. Borretta, Vikramjit Chopra, Alice Y. Chou, Cletus A. D'Souza, Olga Kaspieva, Stéphanie Laprise, Simone C. McInerny, Elodie Portales-Casamar, Magdalena I. Swanson-Newman, Kaelan Wong, George S. Yang, Michelle Zhou, Steven J. M. Jones, Robert A. Holt, Aravind Asokan, Daniel Goldowitz, Wyeth W. Wasserman, and Elizabeth M. Simpson.

## Supplementary Material

**A**

| Ubiquitous Promoter-ORF | Promoter Size (bp) | Plasmid pEMS Number | Animals Evaluated in Brain | % Consistent with Figures | Animals Evaluated in Eye | % Consistent with Figures | Comments |
|---|---|---|---|---|---|---|---|
| CAGGS-icre | 1,723 | 1988 | 8 | 88% | 6 | 83% | Variable in strength |
| CAGGS-icre-WPRE | 1,723 | 1989 | 5 | 80% | 5 | 100% | Variable in strength |
| CAGGS-EmGFP | 1,723 | 2050 | 1 | 100% | 4 | 100% | None |
| CAGGS-EmGFP-WPRE | 1,723 | 2058 | 1 | 100% | 7 | 100% | None |
| smCBA-EmGFP | 961 | 2113 | 2 | 100% | 3 | 100% | None |
| smCBA-EmGFP-WPRE | 961 | 1995 | 2 | 100% | 4 | 100% | None |

**B**

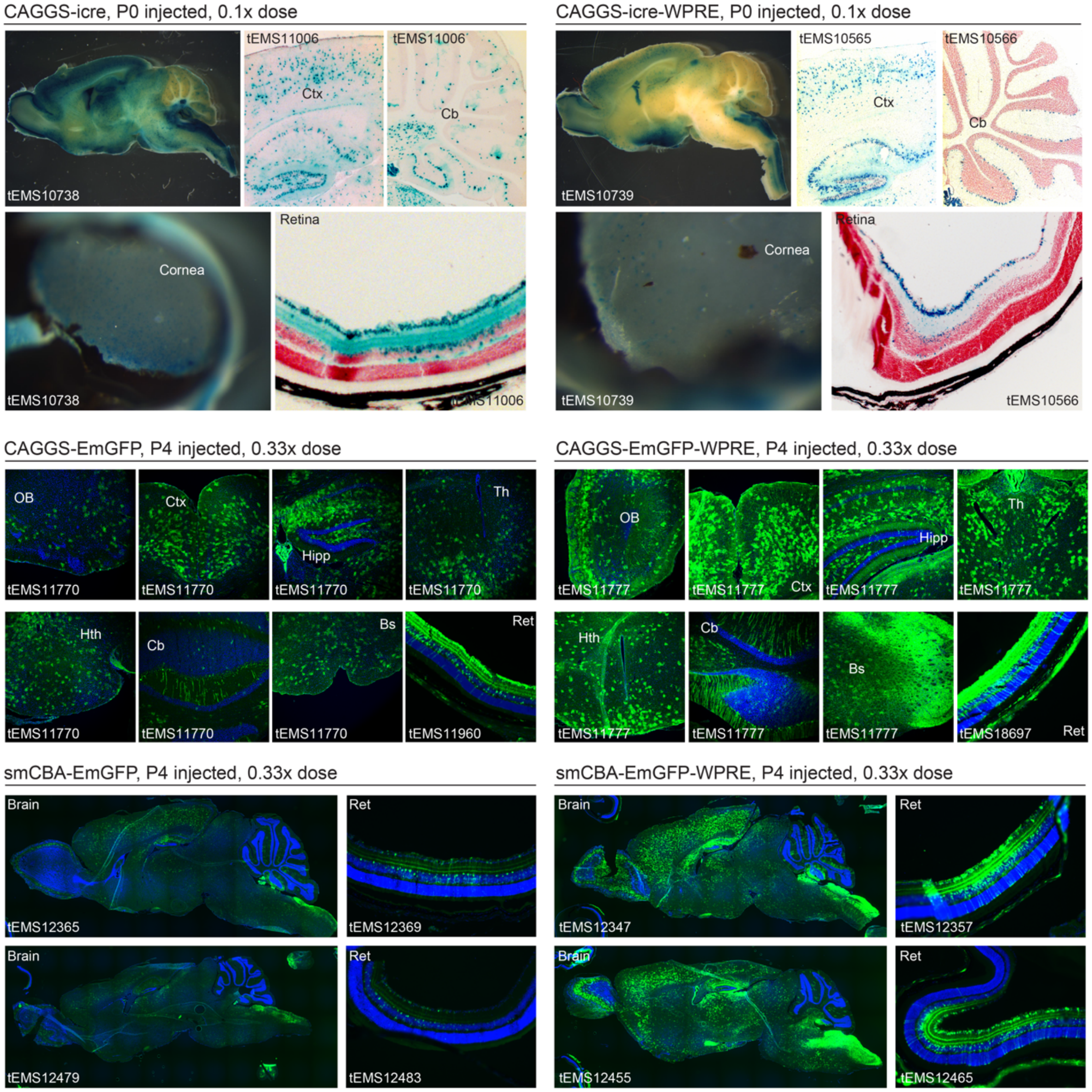


**Figure S1.** CAGGS and smCBA ubiquitous control promoters express throughout all brain regions.

(**a**) Table of ubiquitous control promoter and ORF constructs tested. (**b**) Representative images from each control construct are shown; animals are distinguished by tissue or animal number. Without WPRE (-WPRE) constructs are presented on the left, and with

WPRE (+WPRE) on the right. The CAGGS promoter was assessed with both the icre and EmGFP reporters, whereas smCBA was only assessed with EmGFP. icre constructs were analyzed using X-gal immunohistochemistry and EmGFP constructs with anti-GFP immunofluorescence. Both neurons and glia were transduced. EmGFP, emerald green fluorescent protein; icre, improved cre recombinase; WPRE, woodchuck hepatitis virus post-transcriptional regulatory element. Bs, brainstem; Cb, cerebellum; Ctx, cortex; Hipp, hippocampus; Hth, hypothalamus; OB, olfactory bulb; Ret, retina; tEMS, tissue number; Th, thalamus.

| MiniPromoter-ORF | MiniPromoter Gene | Plasmid pEMS Number | Animals Evaluated in Brain | % Consistent with Figures | Animals Evaluated in Eye | % Consistent with Figures | Animals Evaluated in Muscle | % Consistent with Figures | Comments |
|---|---|---|---|---|---|---|---|---|---|
| Ple34-miniSOG | *CLDN5* | Unassigned | 4 | 100% | NA | NA | NA | NA | None |
| Ple67-EmGFP | *FEV* | 2114 | 6 | 83% | 7 | 71% | NA | NA | Variable in strength in brain (P0 injected) |
| Ple67-EmGFP-WPRE | *FEV* | 2113 | 8 | 100% | 10 | 70% | NA | NA | Variable in strength in brain (P0 injected) |
| Ple94-icre | *GPR88* | 1995 | 8 | 100% | NA | NA | NA | NA | One animal with high background and uneven staining in brain |
| Ple155-EmGFP | *PCP2* | 2116 | 6 | 100% | 8 | 75% | NA | NA | Very weak in eyes (P0 injected) |
| Ple155-EmGFP-WPRE | *PCP2* | 2115 | 6 | 100% | 8 | 100% | NA | NA | Less specific (P0 injected) |
| Ple198-icre | *SLC6A4* | 1997 | 12 | 100% | NA | NA | NA | NA | None |
| Ple251-icre | *C8ORF46* | 1977 | 10 | 100% | 4 | 75% | NA | NA | Consistently negative in cornea |
| Ple251-icre-WPRE | *C8ORF46* | 1978 | 9 | 100% | 4 | 100% | NA | NA | Very strong in cornea |
| Ple253-icre | *PITX3* | 1983 | NA | NA | 4 | 75% | NA | NA | Mostly negative in eye (one weak positive) |
| Ple253-icre-WPRE | *PITX3* | 1984 | NA | NA | 4 | 75% | NA | NA | Mostly positive in eye (one negative in cornea) |
| Ple261-EGFP | *CLDN5* | Unassigned | 4 | 100% | NA | NA | NA | NA | None |
| Ple264-EmGFP | *NR2E1* | 2112 | 12 | 100% | 12 | 33% | NA | NA | P0 injected eyes nearly negative with no clear Müller glia |
| Ple266-icre | *S100B* | 1994 | 6 | 100% | 4 | 25% | NA | NA | Variation in hemisphere expression strength; Müller cells in one mouse |
| Ple267-icre | *UGT8* | 1996 | 8 | 75% | NA | NA | NA | NA | One animal with high dorsal/ventral staining asymmetry |
| Ple301-icre | *TNNT1* | 2026 | NA | NA | NA | NA | 4 | 100% | Tongue and/or quadriceps evaluated |
| Ple302-icre | *DCX* | 2021 | 8 | 75% | 10 | 80% | NA | NA | Some with weak or absent corneal staining |
| Ple303-icre | *NOV* | 2025 | 7 | 100% | 7 | 100% | NA | NA | Positive horizontal cells are rare but present |
| Ple304-icre | *OLIG1* | 1993 | 6 | 83% | NA | NA | NA | NA | None |
| Ple305-icre | *OLIG1* | 2031 | 7 | 100% | NA | NA | NA | NA | Variable in strength |
| hs671-icre | *DPYD-AS1* (intragenic) | 2024 | 6 | 100% | 4 | 100% | NA | NA | Strong staining with some unevenness |
| hs1218-icre | *OTX2-AS1* – *EXOC5* (intergenic) | 2023 | 6 | 100% | NA | NA | NA | NA | Dorsal/ventral staining asymmetry |

**Figure S2.** Consistent restricted expression observed for most positive constructs. Table of 18 MiniPromoter (MiniP) and ORF constructs tested. For each construct, at least 4 animals were assessed in brain, eye, and/or muscle. MiniPs in black text drive icre; MiniPs in green text drive a green fluorescent protein; horizontal lines group MiniP constructs; note that the *CLDN5*-based MiniPs are separated in the table and thus highlighted in pink; bp, base pair; EmGFP; emerald green fluorescent protein; icre, improved cre recombinase; miniSOG, mini Singlet Oxygen Generator; NA, not applicable; ORF, open reading frame; WPRE, woodchuck hepatitis virus post-transcriptional regulatory element.

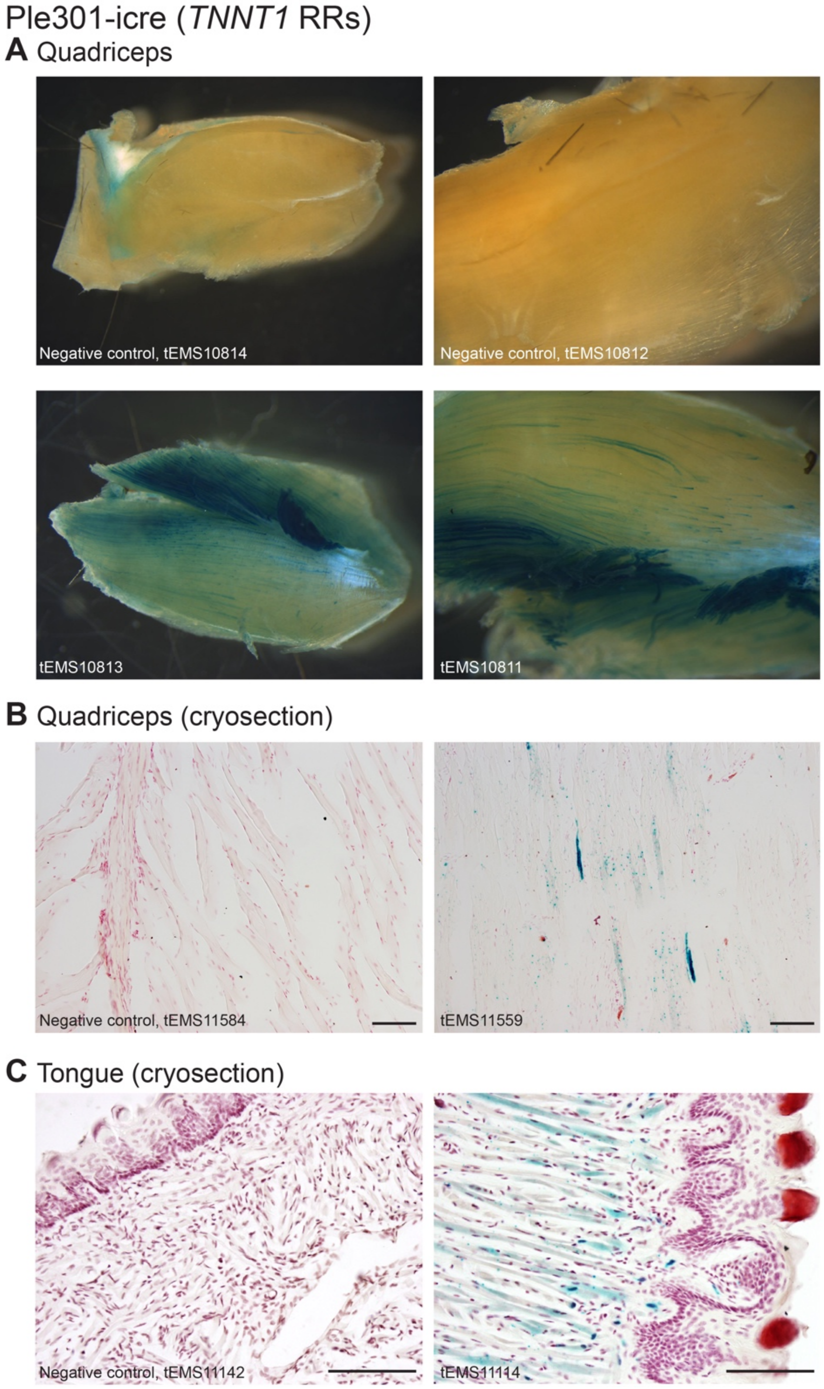


**Figure S3.** The Ple301 (*TNNT1* regulatory regions) MiniP in rAAV drove expression in muscle as expected based on the previously published Ple232 MiniP activity in knock-in mice [1].

(**a**) X-gal staining indicated β-galactosidase activity in the quadriceps muscle, whereas the negative control showed only minimal endogenous staining at the cut site. (**b**) Upon cryosectioning, the individually stained muscle cells are visible. Red, neutral red. (**c**) Similarly, the musculature of the tongue contained β-galactosidase positive cells. Red, neutral red. Each tEMS number indicates a different mouse; two mice are shown per

condition. icre, improved cre recombinase; MiniP, MiniPromoter; RRs, regulatory regions. Blue, β-gal positive.

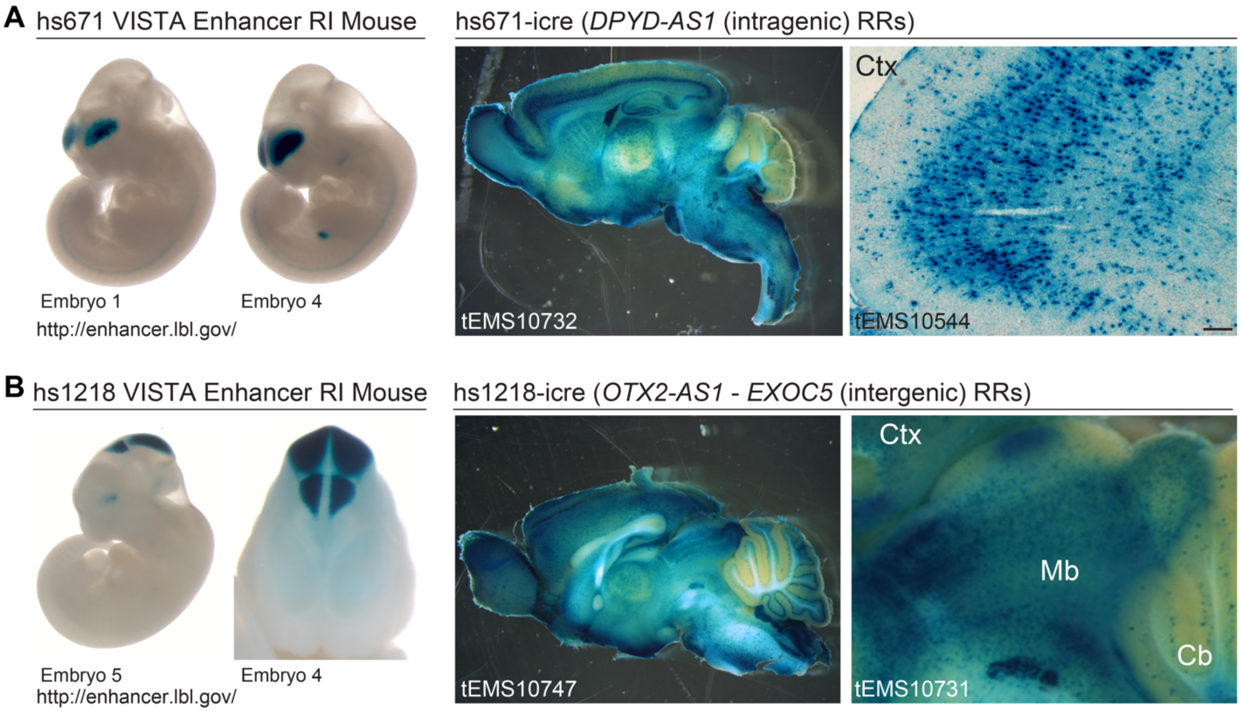


**Figure S4.** Two VISTA enhancers [2] drove unexpectedly broad expression. (**a**) VISTA enhancer hs671 drove highly specific β-galactosidase expression in the developing forebrain of E11.5 mouse embryos after random insertion in the genome (images from http://enhancer.lbl.gov). Using the same promoter design, but now driving icre in rAAV, we identified widespread expression in the adult mouse brain, including extensive staining in the cortex. Red, neutral red. (**b**) VISTA enhancer hs1218 after random-genomic insertion was specific to the midbrain in mouse embryos (images from http://enhancer.lbl.gov). Using this MiniP driving icre in rAAV we observed staining in the midbrain region of the adult mouse brain, but there was also extensive staining throughout the majority of the brain. Each tEMS number indicates a different mouse; two mice are shown per construct. Cb, cerebellum; Ctx, cortex; icre; improved cre recombinase; LBL, Lawrence Berkeley National Laboratory; Mb, midbrain; MiniP, MiniPromoter; RRs, regulatory regions. Blue, β-gal positive.

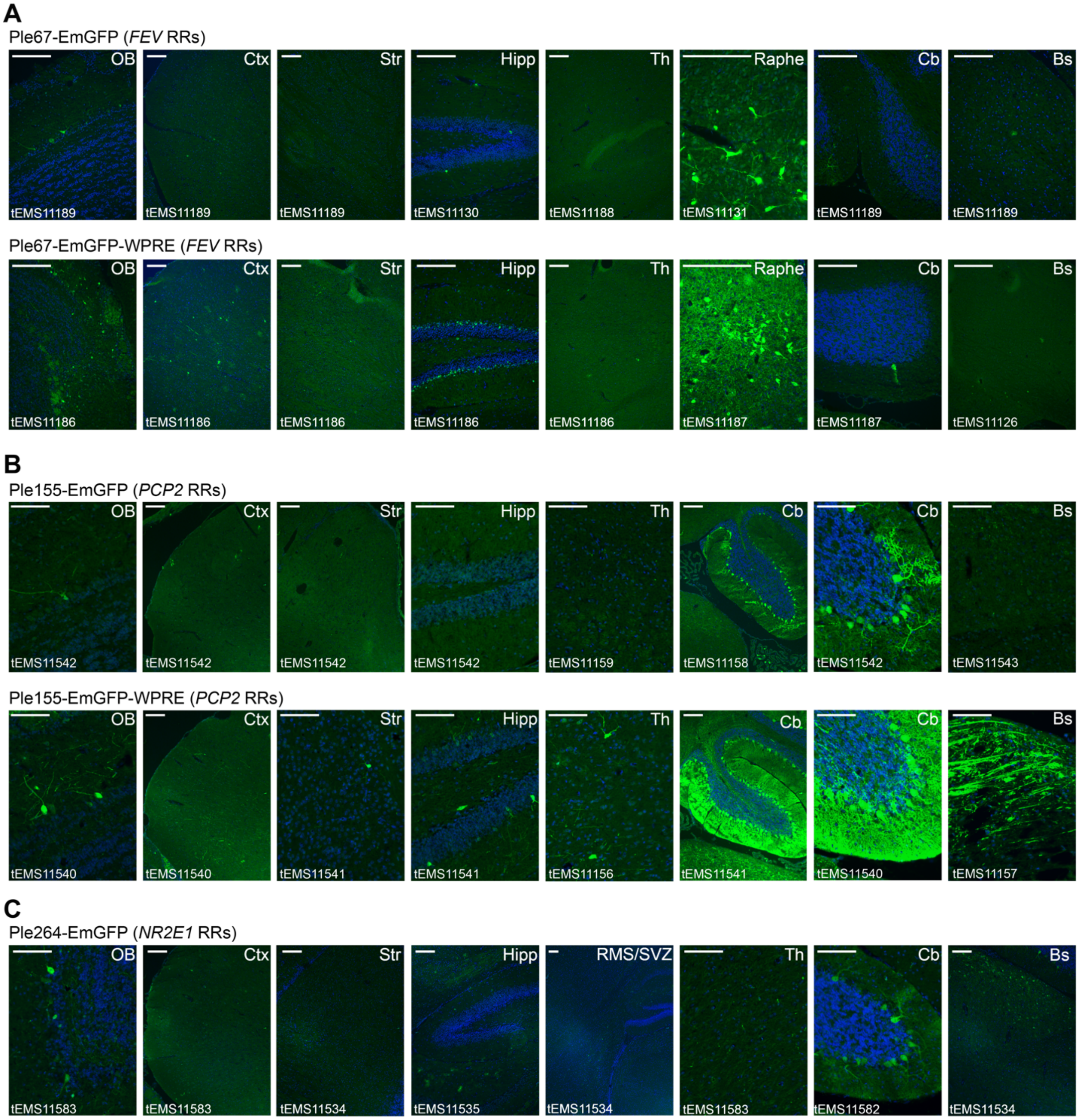


**Figure S5.** MiniPs Ple67 (*FEV* RRs), and Ple155 (*PCP2* RRs) showed strong regional specificity even in the presence of WPRE.

(**a**) Ple67-EmGFP (±WPRE; *FEV* RRs) gave strong expression in the raphe nuclei (green). Shown here are the Dorsal Raphe Nuclei. Only rare scattered cells were positive in other brain regions. (**b**) Ple155-EmGFP (±WPRE; *PCP2* RRs) gave strong expression in the Purkinje cells of the cerebellum (green). Only rare scattered cells were positive in other brain regions. (**c**) Ple264-EmGFP (NR2E1 *RRs*) gave only rare scattered positive cells in any brain region (green). Note that the strong hypothalamus expression of the source Ple140 MiniP, when used in knock-in mice, was absent. Each tEMS number indicates a different mouse. EmGFP, emerald green fluorescent protein; MiniP, MiniPromoter; RRs, regulatory regions; WPRE, woodchuck hepatitis virus post-

transcriptional regulatory element. Bs, brainstem; Cb, cerebellum; Ctx, cortex; Hipp, hippocampus; OB, olfactory bulb; Raphe, raphe nuclei; RMS/SVZ, rostral migratory stream / subventricular zone; RRs, regulatory regions; Str, striatum; Th, thalamus.

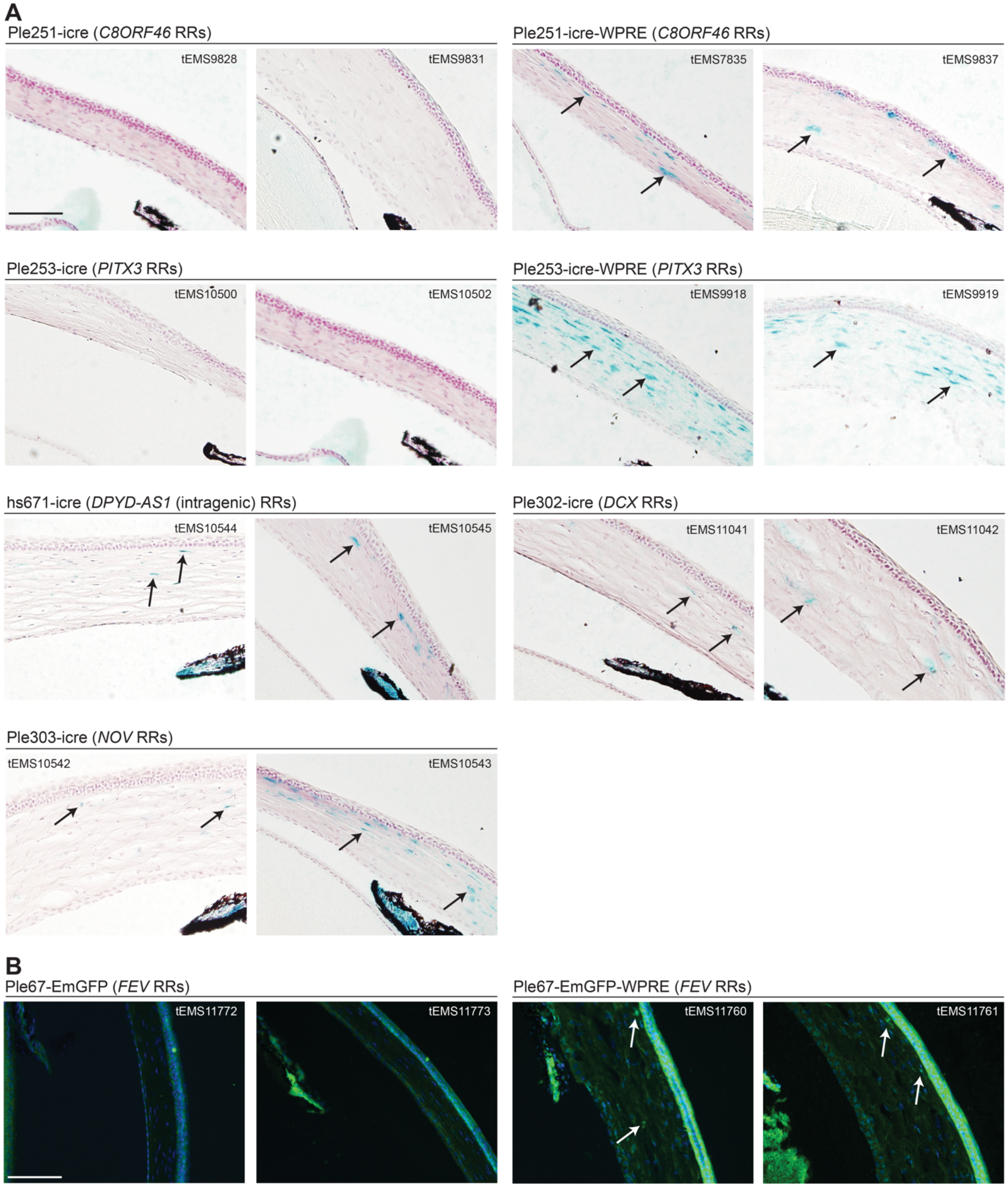


**Figure S6.** Six MiniPs drove expression in the cornea.

(**a**) Ple251, Ple253, hs671, Ple302, and Ple303 drove indirect expression of β-galactosidase detected with X-gal in the corneal stroma, and for Ple251 expression was also detected in the corneal epithelium. For two of these, MiniPs, Ple251 and Ple253, expression detection depended upon the presence of WPRE. Black arrows, β-gal positive (blue); red, neutral red. (**b**) Ple67 drove expression of EmGFP (green) in the corneal stroma. White arrows, EmGFP positive; blue, Hoechst 33342. Each tEMS number indicates a different mouse; two mice are shown per construct. EmGFP, emerald green fluorescent protein; icre, improved cre recombinase; MiniP, MiniPromoter; RRs, regulatory regions; WPRE, woodchuck hepatitis virus post-transcriptional regulatory element. [Scale bars = 100 μm].

# Supplemental References

1. de Leeuw CN, Dyka FM, Boye SL, Laprise S, Zhou M, Chou AY, Borretta LJ, McInerny SC, Banks KG, Portales-Casamar E *et al*: **Targeted CNS delivery using human MiniPromoters and demonstrated compatibility with adeno-associated viral vectors**. *Molecular Therapy - Methods & Clinical Development* 2014, **1**(5):1-15.
2. Visel A, Minovitsky S, Dubchak I, Pennacchio LA: **VISTA Enhancer Browser--a database of tissue-specific human enhancers**. *Nucleic Acids Res* 2007, **35**(Database issue):D88-92.
